# Current Practice and Safety of Invasive Coronary Function Testing: A Nationwide Multicenter Survey in Japan

**DOI:** 10.1016/j.jacadv.2025.102475

**Published:** 2025-12-22

**Authors:** Takeshi Nishi, Michikazu Nakai, Masanobu Ishii, Tetsuya Matoba, Takashi Muramatsu, Kenichi Tsujita, Ken Kozuma, Yoshio Kobayashi, Shiro Uemura, Koichi Kaikita

**Affiliations:** a Department of Cardiovascular Medicine, Chiba University Graduate School Medicine, Chiba, Japan; b Department of Cardiology, Kawasaki Medical School, Kurashiki, Japan; c Clinical Research Support Center, University of Miyazaki Hospital, Miyazaki, Japan; d Faculty of Medicine, Department of Statistics and Data Management, University of Miyazaki, Miyazaki, Japan; e Department of Cardiovascular Medicine, Graduate School of Medical Sciences, Kumamoto University, Kumamoto, Japan; f Department of Cardiovascular Medicine, Graduate School of Medical Sciences, Kyushu University, Fukuoka, Japan; g Department of Cardiology, Fujita Health University Hospital, Toyoake, Japan; h Department of Cardiology, Teikyo University School of Medicine, Tokyo, Japan; i Faculty of Medicine, Division of Cardiovascular Medicine and Nephrology, Department of Internal Medicine, University of Miyazaki, Miyazaki, Japan

**Keywords:** vasospastic angina, coronary spasm, the index microcirculatory resistance, coronary flow reserve, coronary microvascular dysfunction

## Abstract

**Background:**

Coronary vasomotor dysfunction—including vasospastic angina and coronary microvascular dysfunction—is a key mechanism underlying ischemia or myocardial infarction with nonobstructive coronary arteries (INOCA/MINOCA). While invasive coronary function testing (CFT) provides important diagnostic insights, its real-world use and safety—especially in emergency settings—remain poorly defined.

**Objectives:**

The purpose of this study was to assess the current practice, diagnostic yield, and safety of CFT, including acetylcholine (ACh) and ergonovine (ERG) provocation tests and coronary microvascular assessments using coronary flow reserve (CFR) and index of microcirculatory resistance (IMR), in Japan.

**Methods:**

We conducted a nationwide cross-sectional survey of 235 institutions affiliated with the Japanese Cardiovascular Intervention and Therapeutics Society. Data on procedure volume, positivity rates, and complications were collected for tests performed between January 2022 and December 2023.

**Results:**

Among 6,983 provocation tests, 4,700 were ACh (4,327 elective and 373 emergency) and 2,283 were ERG (2,009 elective and 274 emergency). CFR/IMR was measured in 2,192 cases. Positivity rates were 50.9% for ACh and 32.5% for ERG. Major complications occurred in 1.4% of ACh and 0.4% of ERG tests, with no significant difference between emergency and elective settings. Abnormal CFR/IMR findings were more common in emergency cases (45.9% vs 34.8%; *P* < 0.001), while complications remained rare (0.05%).

**Conclusions:**

This large-scale survey confirms the feasibility and safety of invasive CFT, even in emergency settings. These findings support its broader adoption in the diagnostic workup of INOCA and MINOCA.

Coronary vasomotor dysfunction, encompassing vasospastic angina and coronary microvascular dysfunction (CMD), is increasingly recognized as a major cause of myocardial ischemia, even in the absence of obstructive coronary artery disease. These conditions contribute significantly to ischemia with nonobstructive coronary artery disease (INOCA) and myocardial infarction with nonobstructive coronary arteries (MINOCA), both of which are associated with adverse cardiovascular outcomes. While invasive coronary function testing (CFT), including acetylcholine (ACh) and ergonovine (ERG) provocation tests and microvascular assessments such as coronary flow reserve (CFR) and index of microcirculatory resistance (IMR) measurements, plays a crucial role in diagnosing these conditions, its real-world implementation varies widely among institutions.^1^, ^2^, ^3^, ^4^

Previous studies on CFT have primarily originated from expert centers, which limits the generalizability of their findings.^3^, ^4^, ^5^, ^6^, ^7^, ^8^, ^9^, ^10^ Nationwide differences in procedural techniques and standardization have not been thoroughly investigated. Furthermore, the safety of emergency ACh provocation testing has not been sufficiently examined in large-scale studies, despite its potential utility in patients with acute coronary syndromes without obstructive culprit lesions.^11^^,^^12^ This study aims to systematically evaluate the current state of invasive CFT in Japan through a nationwide survey, providing valuable data on procedural trends, diagnostic yield, and associated complications while comparing the international data.

## Methods

A nationwide survey was conducted among 235 institutions affiliated with the Japanese Cardiovascular Intervention and Therapeutics Society and registered in the the National Clinical Database–Japanese Percutaneous Coronary Intervention (NCD J-PCI) registry. The survey was distributed via Google Forms, and responses were collected from March 1, 2024, to September 30, 2024. The questionnaire covered the number of tests performed, diagnostic positivity rates, procedural characteristics, and complication frequencies. The primary safety endpoint was the incidence of major complications associated with CFT including death, ventricular fibrillation, sustained ventricular tachycardia requiring cardioversion, procedure-related myocardial infarction (MI), shock requiring resuscitation, and cardiac tamponade. Secondary outcomes included diagnostic positivity rates and procedural characteristics. The questionnaire was completed by the physician or institutional representative responsible for cardiac catheterization at each center. All institutions were instructed to provide exact counts derived from institutional procedural logs or databases; estimated values were not permitted. All data were collected on a per-procedure basis. Emergency CFT was defined as procedures performed within 24 hours of patient presentation. Although patient-level diagnostic information was not collected in this nationwide survey, in routine Japanese practice emergency CFT is most often performed in patients with suspected MINOCA, acute coronary syndrome without an identifiable culprit lesion, refractory rest angina, and occasionally in resuscitated cardiac arrest patients without obstructive coronary artery disease, in whom coronary spasm is suspected as the underlying mechanism.^11^ The 2023 guideline update from the Japanese cardiovascular societies provide a Class IIb recommendation supporting the use of coronary spasm provocation testing in such acute settings, when alternative causes have been excluded and safety is ensured.^13^ We compared outcomes between emergency procedures and elective procedures (nonemergency). All microvascular measurements were performed using the PressureWire X (Abbott Vascular). Calibration was conducted according to the manufacturer’s instructions, with both pressure and temperature equalization performed before each measurement. Hyperemia was induced most commonly with intracoronary nicorandil or continuous intravenous adenosine, while intracoronary papaverine was used in a minority of centers. A positive finding for coronary spasm on coronary angiography during the ACh- or ERG-induced provocation test was defined as a transient, total, or subtotal focal occlusion (>90% stenosis) of a coronary artery with signs or symptoms of myocardial ischemia (anginal pain and ischemic electrocardiogram changes), or 90% diffuse vasoconstriction involving 2 or more contiguous segments of a coronary artery, according to the diagnostic criteria of the Japanese Circulation Society (JCS) guidelines.^13^^,^^14^ For microvascular assessment, abnormal values were defined as CFR <2.0 and/or IMR ≥25, consistent with established consensus standards.^13^ This study was approved by the Research Ethics Committee of the Cardiovascular Intervention and Therapeutics Society (No. 2024-01). As it was a survey-based study that did not involve individual patient data, the requirement for informed consent was waived.

### Statistical analysis

Continuous data are presented as median (IQR), categorical data as numbers (percentage). For each test modality, positivity and complication rates were reported with 95% CIs. Site-level diagnostic yield was assessed by calculating median (IQR) positivity rates across institutions performing each test. Test of proportions was performed for comparison, and a 2-sided *P* value <0.05 was considered as statistically significant. All analyses were performed using STATA18.5.

## Results

### Overall procedural trends

From January 1, 2022, to December 31, 2023, a total of 186,176 elective coronary angiography and 46,899 emergency coronary angiography were performed among the 235 institutions. During this time, a total of 6,983 provocation tests were conducted among the 235 institutions, including 4,700 (4,327 elective and 373 emergency) ACh tests (Table 1), 2,283 (2,009 elective and 274 emergency) ERG tests (Table 1), and 127 (125 elective and 2 emergency) ACh + ERG tests (Supplemental Table 1). Additionally, 2,192 (elective 2,083 and 109 emergency) CFR and IMR measurements were performed during these procedures (Table 2). The number of CFR/IMR measurements remarkably increased from year 2022 (n = 570) to 2023 (n = 1,622). A total of 210 lactate measurements from aorta and coronary sinus were performed during spasm provocation test for the assessment of objective ischemia.

**Table 1:** Positivity Rate and Complications During ACh and ERG Spasm Provocation Test

ACh Provocation Test	Elective (n = 4,327)	Emergency (n = 373)	*P* Value^a^
Positive provocation test	2,191 (50.6%; 95% CI 49.1-52.1)	203 (54.4%; 95% CI: 49.2-59.6)	0.16
Institutional-level positivity, median [IQR]	53.6% [34.4-71.4]	50.0% [25.0-75.0]	
Major complications	58 (1.34%; 95% CI: 1.02-1.73)	6 (1.60%; 95% CI: 0.59-3.47)	0.68
Death	0	0	
Myocardial infarction	3 (0.07%; 95% CI: 0.01-0.20)	0	0.61
Sustained VT/VF	45 (1.04%; 95% CI: 0.76-1.39)	6 (1.60%; 95% CI: 0.59-3.47)	0.31
Shock	12 (0.28%; 95% CI: 0.14-0.48)	4 (1.07%; 95% CI: 0.29-2.72)	0.012
Cardiac tamponade	1 (0.02%; 95% CI: 0.00-0.13)	0	0.78
ERG provocation test			
Total no.	2009	274	
Positive provocation test	653 (32.5%; 95% CI: 30.5-34.6)	88 (32.1%; 95% CI: 26.6-38.0)	0.90
Institutional-level positivity, median [IQR]	29.5% [13.9-50.0]	35.0% [0.0-50.0]	
Major complications	9 (0.45%; 95% CI: 0.21-0.85)	2 (0.73%; 95% CI: 0.09-2.61)	0.53
Death	0	1 (0.36%; 95% CI: 0.00-2.02)	0.007
Myocardial infarction	0	2 (0.73%; 95% CI: 0.09-2.61)	<0.001
Sustained VT/VF	9 (0.45%; 95% CI: 0.21-0.85)	0	0.27
Shock	0	0	
Cardiac tamponade	0	0	

Ach = acetylcholine; ERG = ergonovine; VF = ventricular fibrillation; VT = ventricular tachycardia.

a *P* value for comparison between elective vs emergency procedures.

**Table 2:** Abnormal Coronary Microvascular Function and Complications Associated With CFR and IMR Measurements During Coronary Spasm Provocation Testing

Variables	Elective (n = 2,083)	Emergency (n = 109)	*P* Value^a^
CFR <2.0	402 (19.3%; 95% CI: 17.6-21.1)	26 (23.9%; 95% CI: 16.2-33.0)	0.25
Institutional-level positivity, median [IQR]	20% [12.7-33.3]	33.3% [20.0-56.8]	
IMR ≥25	658 (31.6%, 95% CI: 29.6-33.6)	49 (45.0%; 95% CI: 35.4-54.8)	<0.001
Institutional-level positivity, median [IQR]	35.0% [23.9-50.0]	51.7% [33.3-75.0]	
Either CFR <2.0 or IMR ≥25	725 (33.9%; 95% CI: 32.8-36.9)	50 (45.9%; 95% CI: 36.3-55.7)	<0.001
Institutional-level positivity, median [IQR]	30.7% [12.5-49.1]	63.6% [40.0-100.0]	
Major complications	1 (0.05%; 95% CI: 0.00-0.27)	0	0.71
Death	0	0	
Myocardial infarction	0	0	
Sustained VT/VF	1 (0.05%, 95% CI: 0.00-0.27)	0	0.71
Shock	0	0	
Cardiac tamponade	0	0	

Abnormal CFR was defined as CFR <2.0; abnormal IMR was defined as IMR ≥25. Joint abnormality refers to the presence of either CFR <2.0 or IMR ≥25.

CFR = coronary flow reserve; IMR = index of microcirculatory resistance; other abbreviations as in Table 1.

a *P* value for comparison between elective vs emergency procedures.

### Procedural characteristics

Procedural characteristics are summarized in Table 3 and Supplemental Tables 2 to 10. The radial artery was the primary vascular access site in the majority of institutions (96%). For spasm provocation testing, the most commonly used catheter size was 5-F (59%), followed by 4-F (40%) and 6-F (<1%). In contrast, for CFR and IMR measurements, 5-F catheters were predominantly used (78%), while 6-F and 4-F were used in 9% and 13% of institutions, respectively. A temporary pacing catheter is routinely used during ACh provocation testing in most of the institutions (93%). The majority of institutions perform ACh provocation testing for both the left coronary artery (LCA) and right coronary artery (RCA) whenever possible, following an incremental dose protocol: 20, 50, and 100 μg for LCA, and 20 and 50 μg for RCA, administered over 20 seconds, in accordance with JCS guidelines.^1^, ^2^, ^3^^,^^14^ Most institutions conduct CFR and IMR measurements exclusively for the left anterior descending artery. The most used hyperemic agents were intracoronary nicorandil (55%), followed by intravenous adenosine (33%) and intracoronary papaverine (7%).

**Table 3:** Acetylcholine Provocation Protocols

	Protocol	Adoption Rate, n/N (%)
LCA ACh dose	JCS guideline protocol (20, 50, 100 μg)	348/401 (86.7%)
	>100 μg in all or selected cases	39/404 (9.7%)
RCA ACh dose	JCS guideline protocol (20, 50 μg)	379/405 (93.6%)
	>50 μg in selected cases	51/408 (12.5%)
ACh injection time	∼20 s (guideline-based)	392/403 (97.2%)
	Longer than 30 s	11/403 (2.7%)
Temporary pacing	Used routinely during ACh provocation	396/424 (93.4%)
Coronary arteries tested	Both coronaries regularly (start LCA)	188/407 (46.2%)
	Both coronaries regularly (start RCA)	90/407 (22.1%)

Data are presented as the number and percentage of institutions reporting each protocol. The denominator represents institution-years with available responses (2022-2023 combined; total N = 472). “No response” was recorded in 71 institution-years (LCA dose), 68 (LCA exceeding dose), 67 (RCA dose), 64 (RCA exceeding dose), 69 (injection time), 48 (pacing), and 65 (coronary arteries tested), as detailed in Supplemental Tables.

JCS = Japanese Circulation Society; LCA = left coronary artery; RCA = right coronary artery; other abbreviation as in Table 1.

### Diagnostic positivity rates and complications

Overall, 2,394 cases (50.9%) were positive for coronary spasm based on the ACh provocation test. In contrast, 705 cases (30.8%) were positive by ERG provocation test, which was significantly lower than the positivity rate of the ACh provocation test (*P* < 0.001). Among cases that underwent sequential ACh and ERG provocation testing, 39 cases (30.7%) were positive on either ACh or ERG testing. For cases that underwent CFR and IMR measurements (n = 2,192), CFR <2.0 was detected in 429 cases (19.5%); IMR ≥25 was noted in 708 cases (32.3%). A total of 775 cases (35.4%) exhibited either CFR <2.0 or IMR ≥25.

Major procedural complications were reported in 64 cases (1.4%) related to the ACh provocation test, 10 cases (0.4%) related to the ERG provocation test, and 2 cases (1.6%) related to sequential ACh and ERG testing. Only one major complication (sustained ventricular tachycardia or ventricular fibrillation) was observed in association with CFR and IMR measurements.

### Comparison of elective vs emergent testing

Spasm positivity rates did not differ significantly between elective and emergency procedures for both ACh provocation (50.6% vs 54.4%; *P* = 0.16) and ERG provocation (32.5% vs 32.1%; *P* = 0.90) (Central Illustration, Table 1). The rate of major complications during the ACh provocation test was similar between elective and emergency settings (1.3% vs 1.6%; *P* = 0.68). However, the rate of cardiogenic shock requiring resuscitation was significantly higher in emergency settings (0.3% vs 1.1%; *P* = 0.012) (Table 1).

**Central Illustration:**
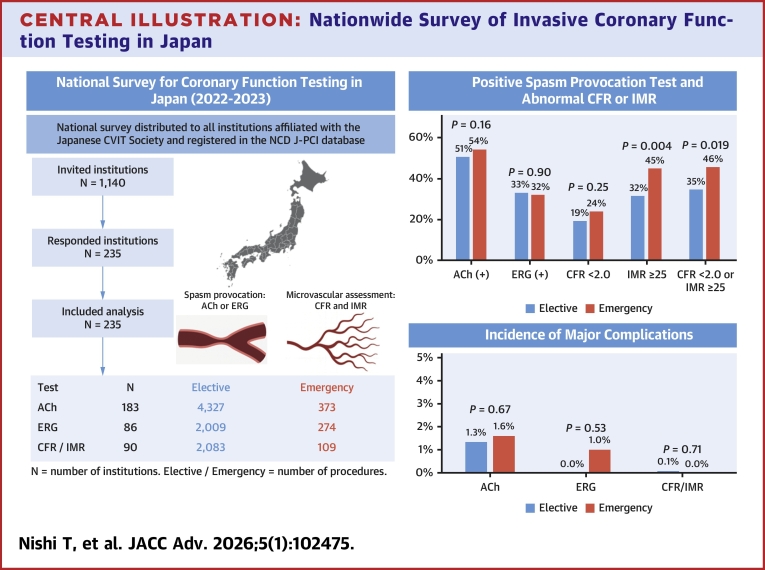
Nationwide Survey of Invasive Coronary Function Testing in Japan Left panel: Flowchart of institutional participation and procedural volumes for coronary function testing reported by 235 Japanese centers from 2022 to 2023. Right panel: Diagnostic yield and incidence of major complications associated with acetylcholine (ACh) and ergonovine (ERG) spasm provocation tests, and coronary flow reserve (CFR) and index of microcirculatory resistance (IMR) measurements, comparing elective vs emergency procedures. Major complications were defined as death, sustained ventricular fibrillation or ventricular tachycardia requiring cardioversion, procedure-related myocardial infarction, cardiogenic shock requiring resuscitation, or cardiac tamponade. Refer to Tables 1 and 2 for full numerical data and CIs. CVIT = Cardiovascular Intervention and Therapeutics; NCD J-PCI = the National Clinical Database–Japanese Percutaneous Coronary Intervention.

Similarly, the rate of major complications during the ERG provocation test did not differ between elective and emergency settings (0.45% vs 0.73%; *P* = 0.53). However, one death and 2 MIs were related to emergency procedures, whereas no deaths or MIs were observed in elective procedures (Table 1).

Abnormal IMR (≥25) was observed more frequently in emergency procedures than in elective procedures (45.0% vs 34.8%; *P* = 0.004). In contrast, CFR <2.0 was observed at similar rates between emergency and elective procedures (23.9% vs 19.3%; *P* = 0.24) (Central Illustration, Table 2). The proportion of cases diagnosed with either CFR <2.0 or IMR ≥25 was significantly higher in emergency procedures compared to elective procedures (45.9% vs 34.8%; *P* < 0.001). The incidence of major complications during CFR and IMR measurements was rare and did not differ significantly between elective and emergency settings (Central Illustration, Table 2).

## Discussion

This nationwide survey provides a comprehensive assessment of the use of invasive CFT across Japan. Our findings confirm the safety of these procedures, including ACh and ERG spasm provocation tests, as well as CFR and IMR measurements. ACh provocation testing remains the primary method for diagnosing vasospastic angina and demonstrates a relatively high positivity rate with an acceptable safety profile. In contrast, ERG testing is less frequently performed, likely due to concerns regarding its lower diagnostic yield and potential safety risks. CFR and IMR measurements were conducted in a subset of cases, revealing a substantial prevalence of CMD.

### Safety of coronary function testing

Prior studies and meta-analyses have consistently reported the safety of ACh and ERG provocation testing, although variability exists in protocols such as injection speed and dosing. Most Japanese institutions adhere to the JCS guideline-recommended approach, administering incremental doses of 20, 50, and 100 μg for the LCA, and 20 and 50 μg for the RCA over 20 seconds.^13^^,^^14^ Notably, the 20-second infusion method is not unique to Japan but is also widely adopted in European and U.S. centers, underscoring its global clinical acceptance and relevance.^3^^,^^8^ Large-scale studies, including the Japanese Coronary Spasm Association registry and recent single-center reports, have shown major complication rates of 0.4 to 2% with ACh or ERG provocation.^5^^,^^6^

Our study expands beyond prior research by assessing the incidence of complications between emergency and elective procedures. To date, multicenter data evaluating the safety of invasive CFT in emergency settings have been extremely limited. Previous single-center studies with smaller patient cohorts have suggested the feasibility of emergency ACh testing;^11^^,^^12^ however, our nationwide survey represents the first large-scale, multicenter investigation to demonstrate its safety and feasibility in real-world emergency settings.

Notably, despite theoretical concerns, our results confirm that the overall rate of major complications remained low in emergency procedures, suggesting that with proper patient selection, emergency ACh testing remains a feasible and valuable diagnostic tool. In ACh provocation testing, cardiogenic shock occurred more frequently in emergency procedures. For ERG provocation testing, there were 2 cases of MI and one death following an emergency procedure, whereas no such events were observed in elective procedures. Patients presenting with unstable chest pain and requiring emergency procedures may exhibit high disease activity, increasing their susceptibility to severe and prolonged coronary spasm. This can lead to critical conditions such as hypotension, cardiogenic shock, and cardiac arrest following provocation testing. Therefore, while emergency CFT remains a valuable diagnostic tool for MINOCA or INOCA presenting with unstable symptoms, clinicians should exercise heightened vigilance when selecting candidates for such procedures. Further investigation into risk stratification methods may help optimize patient outcomes in emergent settings.

Furthermore, our study confirms that CFR and IMR measurements were safe in both elective and emergency settings, further reinforcing their diagnostic value across a wide range of clinical scenarios. Collectively, these findings fill an important knowledge gap and highlight the potential for broader application of emergency CFT in contemporary cardiovascular practice.

### Emerging approaches for CMD assessment

In addition to invasive guidewire-based indices, emerging approaches such as angiography-derived IMR and biomarker-based strategies (eg, metabolomics) are being investigated as complementary tools for CMD assessment. Although still exploratory, these techniques may broaden diagnostic options and support more personalized care in patients with INOCA and MINOCA.

### Clinical implications

During elective testing, a more comprehensive functional assessment including both microvascular and vasospastic evaluation may be appropriate.

In contrast, in emergency settings, given the excellent safety profile of CFR/IMR and the diagnostic importance of ACh provocation, a pragmatic approach in emergencies may be to prioritize microvascular testing as a safe option, while performing vasospasm provocation selectively when clinically appropriate. ERG provocation should be reserved for carefully selected cases, especially in the emergency setting where serious complications tended to cluster. Rather than prescribing a uniform order of testing, these findings support a flexible, patient-tailored approach that balances diagnostic yield and procedural safety.

### Study limitations

Despite its strengths, this study has several limitations. First, the study lacks detailed patient-level covariates, including comorbidities and medication use, as well as long-term follow-up data. Unmeasured case-mix differences—particularly in emergency cases—may have contributed to the higher prevalence of abnormal IMR and could not be fully accounted for in this analysis. Future studies linking survey data with prospective patient-level registries will be essential to validate and extend these findings. Second, the survey-based nature of data collection introduces the potential for reporting bias and variability in institutional practices. Responses were self-reported by each institution, and there might be differences in the interpretation of procedural outcomes and complication rates. In addition, only 235 institutions (20.6%) of the 1,140 invited responded. Because nonresponding institutions may also perform CFT, nonresponse and reporting biases cannot be excluded, and the findings may not fully represent all Japanese centers performing these procedures. Third, the survey did not capture patient-level diagnostic indications for either elective or emergency CFT. Therefore, we could not differentiate between contexts such as MINOCA, unstable angina, or resuscitated cardiac arrest. This absence of diagnostic granularity limits the ability to perform sensitivity analyses restricted to predefined indications and introduces potential heterogeneity in interpreting positivity and safety outcomes. Fourth, although all institutions used the same wire system (PressureWire X, Abbott Vascular), there was variability in hyperemic agents and protocols. While nicorandil and adenosine were commonly used, some centers employed papaverine, and nearly half of institutions did not provide specific protocol details. This heterogeneity may have influenced CFR/IMR values and contributed to intercenter variability. Finally, data were collected on a per-procedure basis; therefore, multiple procedures in the same patient could not be distinguished. Analyses were summarized at the institutional level without adjustment for clustering, as the primary objective was to describe nationwide procedural trends rather than perform inferential comparisons.

## Conclusions

This study represents the large-scale evaluation of invasive CFT in Japan. The findings provide valuable insights into current practices, diagnostic trends, and procedural safety, confirming the feasibility and diagnostic utility of these tests. Emergency testing appears safe with careful patient selection, and linkage with prospective registries will be essential to validate these results in broader real-world settings. Continued efforts are needed to promote comprehensive coronary function assessment to optimize patient care and outcomes.

## Funding support and author disclosures

The authors have reported that they have no relationships relevant to the contents of this paper to disclose.

Perspectives **COMPETENCY IN PATIENT CARE AND PROCEDURAL SKILLS:** Invasive coronary function testing using ACh and ERG provocation, as well as CFR and IMR measurements, is safe and diagnostically effective when performed in both elective and emergency settings. Careful patient selection, particularly in acute cases of suspected INOCA or MINOCA, is essential to minimize risk and guide appropriate management.

**TRANSLATIONAL OUTLOOK:** Future prospective, registry-based studies with detailed patient-level data and long-term follow-up are warranted to determine how invasive coronary function testing impacts prognosis and therapeutic decision-making in patients with suspected coronary vasomotor dysfunction, especially in acute care settings.
