# Supplementary material for: Current Practice and Safety of Invasive Coronary Function Testing: A Nationwide Multicenter Survey in Japan
Source: JACC Adv. 2025 Dec 22;5(1):102475. doi: 10.1016/j.jacadv.2025.102475 (PMC12796537; doi:10.1016/j.jacadv.2025.102475)
Supplement: Supplemental Tables 1-12 [file mmc1.docx]

**Supplementary Materials**

**Supplementary Table 1. Positivity rate and complications during sequential acetylcholine and ergonovine spasm provocation test**

|  | **Elective** | **Emergency** |
| --- | --- | --- |
| Total No. | 125 | 2 |
| Only ACh positive | 13 (10.4%) | 0 |
| Only ERG positive | 15 (12.0%) | 2 (100%) |
| Both ACh and ERG positive | 9 (7.2%) | 0 |
| Either ACh or ERG positive | 37 (29.6%) | 2 (100%) |
| Major complications | 2 (1.6%) | 0 |
| Death | 0 | 0 |
| Myocardial infarction | 0 | 0 |
| Sustained VT/VF | 2 (1.6%) | 0 |
| Shock | 0 | 0 |
| Cardiac tamponade | 0 | 0 |

**Supplementary Table 2. Access site of invasive coronary function test**

| Primary arterial access site | (no response from 9 institutions) |
| --- | --- |
| Radial access | 217/227 |
| Femoral access | 7/227 |
| Other | 3/227 |
| Size of catheter for spasm provocation only | (no response from 10 institutions) |
| 4Fr. | 90/226 |
| 5Fr. | 134/226 |
| 6Fr. | 4/226 |
| 7Fr. | 0 |
| Size of catheter to perform CFR/IMR measurement | (no response from 57 institutions) |
| 5Fr. | 139/179 |
| 6Fr. | 16/179 |
| Other | 24/179 |
| Temporary pacing use during ACh provocation | (no response from 24 institutions) |
| Yes | 198/212 |
| No | 14/212 |
| Temporary pacing primary approach |  |
| Brachial vein | 36/198 |
| Internal jugular vein | 84/198 |
| Femoral vein | 78/198 |

Data represent procedural characteristics in the year 2023 among institutions that responded to the survey. Abbreviations: ACh, acetylcholine; CFR, coronary flow reserve; and IMR, the index of microcirculatory resistance.

**Supplementary Table 3. Coronary Arteries Assessed During Acetylcholine Provocation Testing**

|  | **2022** | **2023** | **Total** |
| --- | --- | --- | --- |
| Both coronary arteries tested whenever possible (starting from the right coronary artery) | 47 (19.92%) | 43 (18.22%) | 90 (19.07%) |
| Both coronary arteries tested whenever possible (starting from the left coronary artery) | 93 (39.41%) | 95 (40.25%) | 188 (39.83%) |
| Test started from the right coronary artery and stopped if positive (if negative, the left coronary artery was also tested) | 32 (13.56%) | 27 (11.44%) | 59 (12.5%) |
| Test started from the left coronary artery and stopped if positive (if negative, the right coronary artery was also tested) | 29 (12.29%) | 29 (12.29%) | 58 (12.3%) |
| Only the left coronary artery or only the right coronary artery was tested | - | - | - |
| Test started based on suspected coronary artery involvement from spontaneous ECG findings | 5 (2.12%) | 7 (2.97%) | 12 (2.54%) |
| No response | 30 (12.71%) | 35 (14.83%) | 65 (13.77%) |

**Supplementary Table 4. Standard Acetylcholine Dosage in the Left Coronary Artery**

| **Administration Method** | **2022** | **2023** | **Total** |
| --- | --- | --- | --- |
| Following JCS guideline protocol (20, 50, 100 μg) | 176 (74.58%) | 172 (72.88%) | 348 (73.73%) |
| 20 μg omitted | 22 (9.32%) | 22 (9.32%) | 44 (9.32%) |
| 50 μg omitted | - | - | - |
| 100 μg only | - | - | - |
| Other | 5 (2.12%) | 4 (1.69%) | 9 (1.91%) |
| No response | 33 (13.98%) | 38 (16.10%) | 71 (15.04%) |
| **Total** | **236** | **236** | **472** |

Abbreviations: JCS, Japanese Circulation Society.

**Supplementary Table 5. Administration of Acetylcholine at Doses Exceeding 100 μg in the Left Coronary Artery**

| **Administration Practice** | **2022** | **2023** | **Total** |
| --- | --- | --- | --- |
| Always administered | 2 (0.85%) | 1 (0.42%) | 3 (0.64%) |
| Administered in selected cases | 17 (7.20%) | 19 (8.05%) | 36 (7.63%) |
| Not administered | 186 (78.81%) | 179 (75.85%) | 365 (77.33%) |
| No response | 31 (13.14%) | 37 (15.68%) | 68 (14.41%) |
| **Total** | **236** | **236** | **472** |

**Supplementary Table 6. Standard Acetylcholine Dosage in the Right Coronary Artery**

| **Administration Practice** | **2022** | **2023** | **Total** |
| --- | --- | --- | --- |
| Following JCS guideline protocol (20, 50 μg) | 189 (80.08%) | 190 (80.51%) | 379 (80.30%) |
| 20 μg omitted | 6 (2.54%) | 5 (2.12%) | 11 (2.33%) |
| 20 μg only | - | - | - |
| Other | 8 (3.39%) | 7 (2.97%) | 15 (3.18%) |
| No response | 33 (13.98%) | 34 (14.41%) | 67 (14.19%) |
| **Total** | **236** | **236** | **472** |

Abbreviations: JCS, Japanese Circulation Society.

**Supplementary Table 7. Administration of Acetylcholine at Doses Exceeding 50 μg**

| **Administration Practice** | **2022** | **2023** | **Total** |
| --- | --- | --- | --- |
| Always administered | 4 (1.69%) | 4 (1.69%) | 8 (1.69%) |
| Administered in selected cases | 21 (8.90%) | 22 (9.32%) | 43 (9.11%) |
| Not administered | 181 (76.69%) | 176 (74.58%) | 357 (75.63%) |
| No response | 30 (12.71%) | 34 (14.41%) | 64 (13.56%) |
| **Total** | **236** | **236** | **472** |

**Supplementary Table 8. Acetylcholine Injection Speed**

| **Injection Speed** | **2022** | **2023** |
| --- | --- | --- |
| Following guideline (administered over ~20 seconds) | 200(84.75%) | 190(80.51%) |
| 20 seconds |  | 2(0.85%) |
| 30 seconds | 4(1.70%) | 3(1.27%) |
| 40 seconds | 1(0.42%) | 1(0.42%) |
| 60 seconds | 1(0.42%) | 1(0.42%) |
| No response | 30(12.71%) | 39(16.53%) |

**Supplementary Table 9. Order of Coronary Spasm Provocation Testing and CFR/IMR Measurement**

| **Order of Measurement** | **2022** | **2023** |
| --- | --- | --- |
| Performed after coronary spasm provocation testing and nitrate administration | 99（41.95％） | 104(44.07%) |
| Performed before coronary spasm provocation testing | 10（4.24％） | 9(3.81%) |
| No CFR and IMR were performed | 32（13.56％） | 30(12.71%) |
| No response | 95（40.25%） | 93(39.41%) |

Abbreviations: CFR, coronary flow reserve; IMR, the index of microcirculatory resistance.

**Supplementary Table 10. Maximum Hyperemic Agents Used**

| **Agent** | **2022** | **2023** |
| --- | --- | --- |
| **Primary Choice** |  |  |
| Continuous intravenous adenosine | 41 (17.37%) | 42 (17.80%) |
| Intracoronary nicorandil | 69 (29.24%) | 70 (29.66%) |
| Intracoronary papaverine | 8 (3.39%) | 9 (3.81%) |
| Other (Not performed) | 3 (1.27%) | 6 (2.54%) |
| No response | 115(48.73%) | 109(46.19%) |
| **Secondary Choice** |  |  |
| Continuous intravenous adenosine | 38 (16.10%) | 37 (15.68%) |
| Intracoronary nicorandil | 42 (17.80%) | 40 (16.95%) |
| Intracoronary papaverine | 14 (5.93%) | 20 (8.47%) |
| Other (Not performed) | 4 (1.7%) | 7 (2.97%) |
| No response | 138(58.47%) | 132(55.93%) |

**Supplementary Table 11. Vessels Assessed for CFR/IMR Measurement**

| **Vessel Assessed** | **2022** | **2023** |
| --- | --- | --- |
| Left anterior descending artery (LAD) only | 98 (41.53%) | 97 (41.10%) |
| LAD and circumflex artery | 2 (0.85%) | 2 (0.85%) |
| Right coronary artery only | - | - |
| All three major coronary arteries whenever possible | 8 (3.39%) | 9 (3.81%) |
| Other (Not performed) | 7 (2.97%) | 9 (3.81%) |
| No response | 121(51.27%) | 119(50.42%) |

**Supplementary Table 12. Participating Institutions**

| **Institution** | **City** | **Prefecture** |
| --- | --- | --- |
| Japanese Red Cross Asahikawa Hospital | Asahikawa | Hokkaido |
| Megumino Hospital | Eniwa | Hokkaido |
| Kitami Red Cross Hospital | Kitami | Hokkaido |
| Kushiro Kōjinkai Memorial Hospital | Kushiro | Hokkaido |
| Engaru Kosei Hospital | Mombetsu District | Hokkaido |
| Steel Memorial Muroran Hospital | Muroran | Hokkaido |
| Obihiro Kyokai General Hospital | Obihiro | Hokkaido |
| Otaru Kyokai Hospital | Otaru | Hokkaido |
| Hokkaido Cardiovascular Hospital | Sapporo | Hokkaido |
| Hokkaido Medical Center | Sapporo | Hokkaido |
| Sapporo Kojinkai Memorial Hospital. | Sapporo | Hokkaido |
| Sapporo Medical University Hospital | Sapporo | Hokkaido |
| Oji General Hospital | Tomakomai | Hokkaido |
| Hirosaki University Hospital | Hirosaki | Aomori |
| Iwate Prefectural Iwai Hospital | Ichinoseki | Iwate |
| Iwate Prefectural Kuji Hospital | Kuji | Iwate |
| Iwate Prefectural Ninohe Hospital | Ninohe | Iwate |
| Iwate Medical University Hospital | Shiwa District | Iwate |
| Ishinomaki Municipal Hospital | Ishinomaki | Miyagi |
| Sendai City Hospital | Sendai | Miyagi |
| Sendai Open Hospital | Sendai | Miyagi |
| Saka General Hospital | Shiogama | Miyagi |
| Nakadori General Hospital | Akita | Akita |
| Akita Cerebrospinal and Cardiovascular Center. | Senshukubotamachi | Akita |
| Hiraka General Hospital | Yokote | Akita |
| Nihonkai General Hospital | Sakata | Yamagata |
| Yamagata University Hospital | Yamagata | Yamagata |
| Takeda General Hospital | Aizuwakamatsu | Fukushima |
| Fukushima Medical University Hospital | Fukushima | Fukushima |
| Wakamatsu Intervention Clinic | Wakamatsu | Fukushima |
| Hitachi General Hospital | Hitachi | Ibaraki |
| Moriya Daiichi General Hospital | Moriya | Ibaraki |
| Ibaraki Seinan Medical Center Hospital | Sashima District | Ibaraki |
| Tsukuba Memorial Hospital | Tsukuba | Ibaraki |
| Tsukuba Central Hospital | Ushiku | Ibaraki |
| Ushiku Aiwa General Hospital | Ushiku | Ibaraki |
| International University of Health and Welfare Hospital | Nasushiobara | Tochigi |
| Dokkyo Medical University Hospital | Shimotsuga | Tochigi |
| Gunma University Hospital | Maebashi | Gunma |
| Kitakanto Cardiology Hospital | Shibukawa | Gunma |
| Ageo Central General Hospital | Ageo | Saitama |
| Gyoda General Hospital | Gyoda | Saitama |
| Saitama Medical University International Medical Center | Hidaka | Saitama |
| Sekishindo Hospital | Kawagoe | Saitama |
| Kawaguchi Cardiology and Respiratory Hospital | Kawaguchi | Saitama |
| Kitasato University Medical Center | Kitamoto | Saitama |
| Saitama Prefectural Cardiovascular and Respiratory Center | Kumagaya | Saitama |
| Jichi Medical University Saitama Medical Center | Saitama | Saitama |
| Saitama Sekishinkai Hospital | Sayama | Saitama |
| Tokorozawa Heart Center | Tokorozawa | Saitama |
| Chiba University Hospital | Chiba | Chiba |
| Chiba Rosai Hospital | Ichihara | Chiba |
| Kameda Medical Center | Kamogaaw | Chiba |
| Tokyo Bay Urayasu Ichikawa Medical Center | Urayasu | Chiba |
| Juntendo University Hospital | Bunkyo | Tokyo |
| Mitsui Memorial Hospital | Chiyoda | Tokyo |
| Tokyo Teishin Hospital | Chiyoda | Tokyo |
| St. Luke’s International Hospital | Chuo | Tokyo |
| Tokyo Rinkai Hospital | Edogawa | Tokyo |
| Fuchu Keijinkai Hospital | Fuchu | Tokyo |
| Tokyo Metropolitan Tama Medical Center | Fuchu | Tokyo |
| Tama Hokubu Medical Center | Higashimurayama | Tokyo |
| Higashiyamato Hospital | Higashiyamato | Tokyo |
| Itabashi Chuo Medical Center | Itabashi | Tokyo |
| Teikyo University Hospital | Itabashi | Tokyo |
| Tokyo Metropolitan Institute for Geriatrics and Gerontology | Itabashi | Tokyo |
| IMS Katsushika Heart Center | Katsushika | Tokyo |
| Jikei University Katsushika Medical Center | Katsushika | Tokyo |
| Akabane Central General Hospital | Kita | Tokyo |
| Machida Municipal Hospital | Machida | Tokyo |
| National Hospital Organization Tokyo Medical Center | Meguro | Tokyo |
| International University of Health and Welfare Mita Hospital | Minato | Tokyo |
| Kitasato University Kitasato Institute Hospital | Minato | Tokyo |
| The Cardiovascular Institute | Minato | Tokyo |
| Japanese Red Cross Musashino Hospital | Musashino | Tokyo |
| Tokyo Metropolitan Police Hospital | Nakano | Tokyo |
| Juntendo University Nerima Hospital | Nerima | Tokyo |
| Ome Medical Center | Ome | Tokyo |
| Japanese Red Cross Medical Center | Shibuya | Tokyo |
| Tokyo Metropolitan Hiroo Hospital | Shibuya | Tokyo |
| Showa Medical University Hospital | Shinagawa | Tokyo |
| Tokyo Medical University Hospital | Shinjuku | Tokyo |
| Tokyo Women's Medical University Hospital | Shinjuku | Tokyo |
| Tokyo Metropolitan Ohkubo Hospital | Shinjyuku | Tokyo |
| Kawakita General Hospital | Suginami | Tokyo |
| National Disaster Medical Center | Tachikawa | Tokyo |
| Tachikawa Sogo Hospital | Tacihkawa | Tokyo |
| Nippon Medical School Tamanagayama Hospital | Tama | Tokyo |
| Atsugi City Hospital | Atsugi | Kanagawa |
| Japanese Red Cross Hadano Hospital | Hadano | Kanagawa |
| Tokai University Hospital | Isehara | Kanagawa |
| Shin-Yurigaoka General Hospital | Kawasaki | Kanagawa |
| St. Marianna Medical University Hospital | Kawasaki | Kanagawa |
| Takatsu Central General Hospital | Kawasaki | Kanagawa |
| Kitasato University Hospital | Sagamihara | Kanagawa |
| Sagamihara Kyodo Hospital | Sagamihara | Kanagawa |
| Sagamihara National Hospital | Sagamihara | Kanagawa |
| Showa Medical University Fujigaoka Hospital | Yokohama | Kanagawa |
| SHOWA Medical University Northern Yokohama Hospital | Yokohama | Kanagawa |
| Yokohama Chuo Hospital | Yokohama | Kanagawa |
| Yokohama City University Medical Center | Yokohama | Kanagawa |
| Yokohama Municipal Citizen's Hospital | Yokohama | Kanagawa |
| Yokohama-Minami Kyosai Hospital | Yokohama | Kanagawa |
| Joetsu General Hospital | Joetsu | Nigata |
| Tonami General Hospital | Tonami | Toyama |
| Toyama City Hospital | Toyama | Toyama |
| Toyama Prefectural Central Hospital | Toyama | Toyama |
| Ishikawa Prefectural Central Hospital | Kanazawa | Ishikawa |
| Fukui Saiseikai Hospital | Fukui | Fukui |
| University of Yamanashi Hospital | Chuo | Yamanashi |
| Kofu Municipal Hospital | Kofu | Yamanashi |
| Shinshu University | Matsumoto | Nagano |
| Nagano Chuō Hospital | Nagano | Nagano |
| Hokushin General Hospital | Nakano | Nagano |
| Gifu Prefectural General Medical Center | Gifu | Gifu |
| Gifu University Hospital | Gifu | Gifu |
| Hamamatsu University Hospital | Hamamatsu | Shizuoka |
| Iwata General Hospital | Iwata | Shizuoka |
| Numazu City Hospital | Numazu | Shizuoka |
| Shimada General Medical Center | Shimada | Shizuoka |
| National Hospital Organization Shizuoka Medical Center | Sunto District | Shizuoka |
| Japanese Red Cross Nagoya Daini Hospital | Nagoya | Aichi |
| Kyōritsu General Hospital | Nagoya | Aichi |
| Nagoya City University East Medical Center | Nagoya | Aichi |
| Nagoya City University West Medical Center | Nagoya | Aichi |
| Nagoya University Hospital | Nagoya | Aichi |
| Tosei General Hospital | Seto | Aichi |
| Toyota Kosei Hospital | Toyota | Aichi |
| Toyota Memorial Hospital | Toyota | Aichi |
| Japanese Red Cross Ise Hospital | Ise | Mie |
| Matsusaka Chuō General Hospital | Matsusaka | Mie |
| Saiseikai Matsusaka General Hospital | Matsusaka | Mie |
| Nabari Municipal Hospital | Nabari | Mie |
| Koto Memorial Hospital | Higashiomi | Shiga |
| Hikone Municipal Hospital | Hikone | Shiga |
| Biwako Ōhashi Hospital | Otsu | Shiga |
| Shiga University of Medical Science Hospital | Otsu | Shiga |
| Saiseikai Shiga Prefecture Hospital | Ritto | Shiga |
| Japanese Red Cross Kyoto Daiichi Hospital | Kyoto | Kyoto |
| Koseikai Takeda Hospital | Kyoto | Kyoto |
| Kyoto City Hospital | Kyoto | Kyoto |
| Mitsubishi Kyoto Hospital | Kyoto | Kyoto |
| Rakusai Newtown Hospital | Kyoto | Kyoto |
| Rakuwakai Marutamachi Hospital | Kyoto | Kyoto |
| Rakuwakai Otowa Hospital | Kyoto | Kyoto |
| Maizuru Kyosai Hospital | Maizuru | Kyoto |
| Kayashima Ikuno Hospital | Kadoma | Osaka |
| Chibune General Hospital | Osaka | Osaka |
| Medical Research Institute KITANO HOSPITAL | Osaka | Osaka |
| NHO Osaka National Hospital | Osaka | Osaka |
| Osaka General Medical Center | Osaka | Osaka |
| Osaka Red Cross Hospital | Osaka | Osaka |
| Nippon Life Saiseikai Nippon Life Hospital | Osaka | Osaka |
| Yodogawa Christian Hospital | Osaka | Osaka |
| Saiseikai Senri Hospital | Suita | Osaka |
| Toyonaka Municipal Hospital | Toyonaka | Osaka |
| Yao Municipal Hospital | Yao | Osaka |
| Ako City Hospital | Ako | Hyogo |
| Amagasaki General Medical Center | Amagasaki | Hyogo |
| Hyogo Prefectural Harima-Himeji General Medical Center | Himeji | Hyogo |
| Tsukazaki Hospital | Himeji | Hyogo |
| Kobe Century Memorial Hospital | Kobe | Hyogo |
| Kobe Rosai Hospital | Kobe | Hyogo |
| Hyogo Medical University Hospital | Nishimiya | Hyogo |
| Higashi Takarazuka Satoh Hospital | Takarazuka | Hyogo |
| Takarazuka City Hospital | Takarazuka | Hyogo |
| Takarazuka Hospital | Takarazuka | Hyogo |
| Ikoma City Hospital | Ikoma | Nara |
| Nara Medical University Hospital | Kashihara | Nara |
| Shingu Municipal Medical Center | Shingu | Wakayama |
| Shimane University Hospital | Izume | Shimane |
| Japanese Red Cross Masuda Hospital | Masuda | Shimane |
| Kawasaki Medical School | Kurashiki | Okayama |
| Kurashiki Central Hospital | Kurashiki | Okayama |
| Okayama City Civic Hospital | Okayama | Okayama |
| Higashihiroshima Medical Center | Higashihiroshima | Hiroshima |
| Hiroshima City North Medical Center Asa Citizens Hospital | Hiroshima | Hiroshima |
| Hiroshima Prefectural Hospital | Hiroshima | Hiroshima |
| Hiroshima University Hospital | Hiroshima | Hiroshima |
| JR Hiroshima Hospital | Hiroshima | Hiroshima |
| Tsuchiya General Hospital | Hiroshima | Hiroshima |
| Yamaguchi Rosai Hospital | Sanyoonoda | Yamaguchi |
| Shimonoseki City Hospital | Shimonoseki | Yamaguchi |
| Yamaguchi University Hospital | Ube | Yamaguchi |
| Tokushima University Hospital | Tokushima | Tokushima |
| Mitoyo General Hospital | Kanonji | Kagawa |
| Kagawa University Hospital | Kita District | Kagawa |
| Kagawa Prefectural Central Hospital | Takamatsu | Kagawa |
| Takamatsu Hospital | Takamatsu | Kagawa |
| HITO Hospital | Shikokuchuo | Ehime |
| Uwajima City Hospital | Uwajima | Ehime |
| Aki General Hospital | Akita | Kochi |
| Chikamori Hospital | Kochi | Kochi |
| Hosogi Hospital | Kochi | Kochi |
| Hata Kenmin Hospital | Sukumo | Kochi |
| Saiseikai Wakayama Hospital | Wakayama | Wakayama |
| Wakayama Medical University Hospital | Wakayama | Wakayama |
| Kyushu University Hospital | Fukuoa | Fukuoka |
| Chihaya Clinics | Fukuoka | Fukuoka |
| Fukuoka City Hospital | Fukuoka | Fukuoka |
| Fukuoka Kinen Hospital | Fukuoka | Fukuoka |
| Fukuoka Sanno Hospital | Fukuoka | Fukuoka |
| Fukuoka University Nishijin Hospital | Fukuoka | Fukuoka |
| Saiseikai Fukuoka General Hospital | Fukuoka | Fukuoka |
| Munakata Suikokai General Hospital | Fukutsu | Fukuoka |
| Iizuka Hospital | Iizuka | Fukuoka |
| Hagiwara Central Hospital | Kitakyushu | Fukuoka |
| Kitakyushu City Yahata Hospital | Kitakyushu | Fukuoka |
| Steel Memorial Yawata Hospital | Kitakyushu | Fukuoka |
| University of the Occupational and Environmental Health | Kitakyushu | Fukuoka |
| Kurume University | Kurume | Fukuoka |
| Takagi Hospital | Okawa | Fukuoka |
| Omuta Tenryo Hospital | Omuta | Fukuoka |
| Sugi Gastroenterology Hospital | Omuta | Fukuoka |
| Nagasaki Memorial Hospital | Nagasaki | Nagasaki |
| Nagasaki University Hospital | Nagasaki | Nagasaki |
| Amakusa Medical Center | Amakusa | Kumamoto |
| JCHO Hitoyoshi Medical Center | Hitoyoshi | Kumamoto |
| Kumamoto Chuo Hospital | Kumamoto | Kumamoto |
| Kumamoto University Hospital | Kumamoto | Kumamoto |
| Saiseikai Kumamoto Hospital | Kumamoto | Kumamoto |
| Kumamoto Kenhoku Hospital | Tamana | Kumamoto |
| Tenshindo Hetsugi Hospital | Oita | Oita |
| NHO Oita Medical Center | Yokota | Oita |
| Miyakonojoshi Gunishikai Clinics | Miyakonojo | Miyazaki |
| Miyazaki Medical Association Hospital | Miyazaki | Miyazaki |
| University of Miyazaki | Miyazaki | Miyazaki |
| Nobeoka Hospital | Nobeoka | Miyazaki |
| Oshima Hospital | Amami | Kagoshima |
| Kagoshima City Hospital | Kagoshima | Kagoshima |
| Kagoshima University Hospital | Kagoshima | Kagoshima |
| Okinawa Prefectural Miyako Hospital | Miyakojima | Okinawa |
| Ohama Dai-ichi Hospital | Naha | Okinawa |
| Okinawa Chubu Hospital | Uruma | Okinawa |

This table lists the participating institutions based on available information, organized by prefecture. A total of 235 institutions contributed data to the nationwide survey on invasive coronary function testing. One hospital was not listed.
